# Immunotherapy Based on Immune Checkpoint Molecules and Immune Checkpoint Inhibitors in Gastric Cancer–Narrative Review

**DOI:** 10.3390/ijms25126471

**Published:** 2024-06-12

**Authors:** Agata Poniewierska-Baran, Karolina Sobolak, Paulina Niedźwiedzka-Rystwej, Paulina Plewa, Andrzej Pawlik

**Affiliations:** 1 Center of Experimental Immunology and Immunobiology of Infectious and Cancer Diseases, University of Szczecin, 71-417 Szczecin, Poland; agata.poniewierska-baran@usz.edu.pl (A.P.-B.); paulina.niedzwiedzka-rystwej@usz.edu.pl (P.N.-R.); 2 Institute of Biology, University of Szczecin, 71-412 Szczecin, Poland; 3 Department of Physiology, Pomeranian Medical University, 70-111 Szczecin, Poland; 4 Students Research Club of Immunobiology of Infectious and Cancer Diseases “NEUTROPHIL”, University of Szczecin, 71-417 Szczecin, Poland; 232901@stud.usz.edu.pl (K.S.); paulina.plewa@op.pl (P.P.)

**Keywords:** gastric cancer, immunotherapy, immune checkpoint molecules, PD-1, PD-L1, CTLA-4, immune checkpoint inhibitors

## Abstract

Due to its rapid progression to advanced stages and highly metastatic properties, gastric cancer (GC) is one of the most aggressive malignancies and the fourth leading cause of cancer-related deaths worldwide. The metastatic process includes local invasion, metastasis initiation, migration with colonisation at distant sites, and evasion of the immune response. Tumour growth involves the activation of inhibitory signals associated with the immune response, also known as immune checkpoints, including PD-1/PD-L1 (programmed death 1/programmed death ligand 1), CTLA-4 (cytotoxic T cell antigen 4), TIGIT (T cell immunoreceptor with Ig and ITIM domains), and others. Immune checkpoint molecules (ICPMs) are proteins that modulate the innate and adaptive immune responses. While their expression is prominent on immune cells, mainly antigen-presenting cells (APC) and other types of cells, they are also expressed on tumour cells. The engagement of the receptor by the ligand is crucial for inhibiting or stimulating the immune cell, which is an extremely important aspect of cancer immunotherapy. This narrative review explores immunotherapy, focusing on ICPMs and immune checkpoint inhibitors in GC. We also summarise the current clinical trials that are evaluating ICPMs as a target for GC treatment.

## 1. Introduction

Gastric cancer (GC) is the fourth leading cause of cancer-related deaths and the fifth most prevalent type of cancer worldwide. By 2040, the annual burden of GC is projected to increase to ~1.8 million new cases and ~1.3 million deaths [1]. Most cases are reported in Asia (75%), where it is considered to be a serious health problem. GC occurs most often after the age of 50 years, with approximately three fourth of cases in both sexes occurring in the elderly population (over 65 years of age). Therefore, the risk of developing GC is correlated with age. GC has a relatively poor prognosis, with a 5-year survival rate of only about 36% according to data from the Surveillance, Epidemiology, and End Results (SEER) database. Lauren classification is the most common system used to classify GC; it recognises the intestinal and diffuse types [2]. There are two topographically, epidemiologically, and etiologically different subtypes of GC: cardia (proximal) and non-cardia (distal) [3]. Depending on the molecular cause, four subtypes can be identified: Epstein–Barr virus (EBV) positive, microsatellite instability, chromosomal instability, and genomically stable tumours [4].

**Figure 1:**
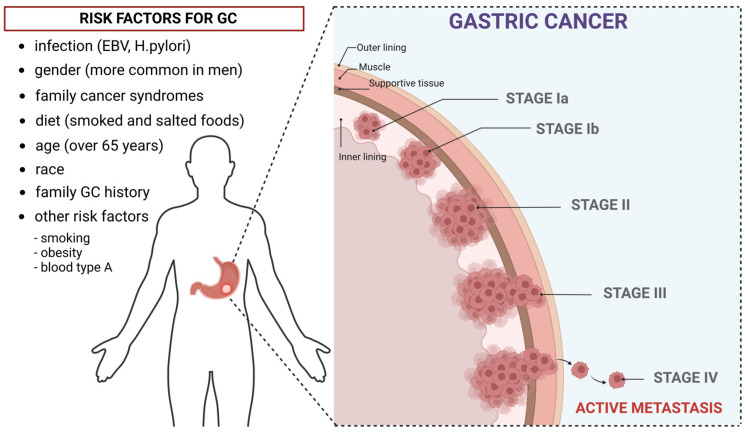
The risk factors and the initiation process of gastric cancer (GC). This figure was created under the bioRENDER license.

GC risk factors (Figure 1) include obesity, excessive alcohol consumption, smoking, Heliobacter pylori infections, and poor dietary habits. Diets rich in salt, N-nitroso compounds [5], smoked foods, and red meat [6] increase the risk of GC. Interestingly, fresh fruit and vegetable consumption decreases the risk of developing GC [7]. Obesity is strongly associated with GC; it entails many changes in the body that predispose an individual to GC, including hormonal imbalance, increased inflammation, and reflux [8]. According to the World Health Organization (WHO), H. pylori is considered a carcinogen. Moreover, EBV can also lead to GC [9,10]. Apart from environmental factors, which are considered the major contributors to GC development, genetic predispositions are also important. The most common mutations related to GC occur in the CDH1 gene; in addition, familial aggregation is relatively uncommon for this neoplasm [11]. 

GC often displays no symptoms, especially in the early stages. The usual symptoms are nonspecific and include abnormal bowel movements, nausea, vomiting, haematemesis (in 10%–15% of cases), weight loss, decreased appetite, and abdominal pain [12]. In most countries, early-stage detection rates are low, a factor that drastically decreases 5-year survival rates. By contrast, in Japan GC is detected relatively early due to precautionary screenings, and the 5-year survival rates are remarkably longer [13]. The peritoneum is the most common site of GC metastasis, and, despite radical surgery, the average survival time is only 6 months after diagnosis of peritoneal metastases, compared with 14 months for GC without peritoneal metastases. This shows the importance of early GC diagnosis.

Prevention of GC is the key; improving diet, quitting bad habits (smoking and drinking), and regular medical check-ups can therefore greatly decrease the chance of developing GC or at least improve the outcomes. Some GC biomarkers are detectable in blood serum, including carcinoembryonic antigen (CEA), cancer antigen CA19-9, CA72-4, and CA125. There are several biomarkers in liquid biopsies (obtained non-invasively), including circulating tumour cells, long non-coding RNAs, cell-free DNA, microRNAs, and exosomes. Assessment of these biomarkers may help to identify those at risk of developing GC and/or facilitate an early diagnosis of this disease [14]. Several molecular GC biomarkers have been extensively explored. The main signatures of GC development include human epidermal growth factor receptor 2 (HER2) expression as well as regulatory factors of apoptosis and the cell cycle, including proteins responsible for cell membrane properties, multidrug resistance, and microsatellite instability [15]. 

## 2. Standard Treatment Strategies for Gastric Cancer

The standard and most effective treatment for GC is gastrectomy, which involves resection of all cancerous and surrounding tissues, usually along with the nearest lymph nodes. The extent of stomach resection is determined for each individual patient based on disease progression and the aim of surgery—curative, palliative, or reduction [13,16]. Before commencing surgical treatment, laparoscopy is performed to gain a more accurate assessment of the extent of the tumour and whether it can be treated surgically. Immunotherapy could be used as neoadjuvant preceding the surgery or as an adjunct therapy. Both methods increase patient survival.

Chemotherapy can shrink tumours and prolong the patient’s life. It is mainly used for patients with GC that cannot be resected, in advanced stages of the disease, and/or when there is metastasis. All patients are evaluated for eligibility for this treatment and to determine which drugs are to be used for the best results. The most-used anticancer drugs for GC treatment include cisplatin, oxaliplatin, 5-fluorouracil (5-FU), and paclitaxel, among others. Many combined therapy regimes are developed and widely used such as SOX (S-1 and oxaliplatin), FOLFOX (5-FU/levofolinate calcium and oxaliplatin), and FOLFIRINOX (fluorouracil, leucovorin, oxaliplatin, and irinotecan) [13,16].

## 3. Immunotherapy in Gastric Cancer

In most cases, cancer immunotherapy plays a complementary role to surgery, chemotherapy, or radiotherapy. However, surgical removal of the GC tumour is not always possible. In such cases, patients are treated with chemotherapy, immunotherapy, or broader, a molecularly targeted treatment as a part of the drug programme. Such modern medicine has increased the chances of survival and recovery.

Cancer immunotherapy involves activating the immune system, which has many natural anti-cancer defence mechanisms. The first immunocompetent drug, an anti-cytotoxic T4 lymphocyte antigen (CTLA-4) antibody (ipilimumab), was created based on research by Nobel Prize winners James P. Allison and Tasuku Honjo [17] and registered in 2011. It was a breakthrough discovery. Currently, immunotherapy plays a practical role in the treatment of many malignancies, including GC. There are two forms of immunotherapy: Passive immunotherapy uses monoclonal antibodies (mAbs) generated outside of the body to target cancer cells. This is the most frequently used method of cancer immunotherapy in clinical practice; it can enhance the host’s immune response or inhibit tumour development by blocking cancer growth factors. By contrast, active immunotherapy focuses on boosting the body’s immune response against cancer cells (e.g., vaccinations and chimeric antigen receptors) [18]. The effect of immunotherapy is more selective, offering greater protection of healthy tissues with fewer side effects. For certain groups of patients with cancer, immunological treatment works spectacularly, and the effects of treatment last for many years, even in the case of advanced cancers. 

Regarding gastrointestinal cancers, immunotherapy has become the first-line treatment for microsatellite-instability-high (MSI-H) late-stage colorectal cancer and the first-line treatment for late-stage GC, albeit combined with chemotherapy and HER2-targeted drugs (in HER2-positive patients, approximately 20% of patients with GC). This combination has shown significant efficacy and has enhanced long-term patient survival by enhancing antibody-dependent, cell-mediated cytotoxicity against tumour cells via natural killer (NK) cells. HER2 is a receptor tyrosine kinase that belongs to the human epidermal growth factor receptor (EGFR) family of tyrosine kinase receptors [19]. Unfortunately, how to qualify cancers as HER2 positive remains a debated topic. Another problem that may make immunotherapy impossible or reduce its effectiveness is the stage of advancement and the presence of GC metastases. Interestingly, Liu et al. [20] showed that immunotherapy is less effective in patients with GC with liver metastases compared with those without liver metastasis.

Currently, immunotherapy also focuses on the use of drugs or combinations of drugs and on targeting immune checkpoints. Since 2011, when ipilimumab was first approved for the treatment of BRAF-negative metastatic melanoma, studies of immune checkpoint inhibitors (ICIs) have become very popular.

### 3.1. Immune Checkpoint Molecules

**Figure 2:**
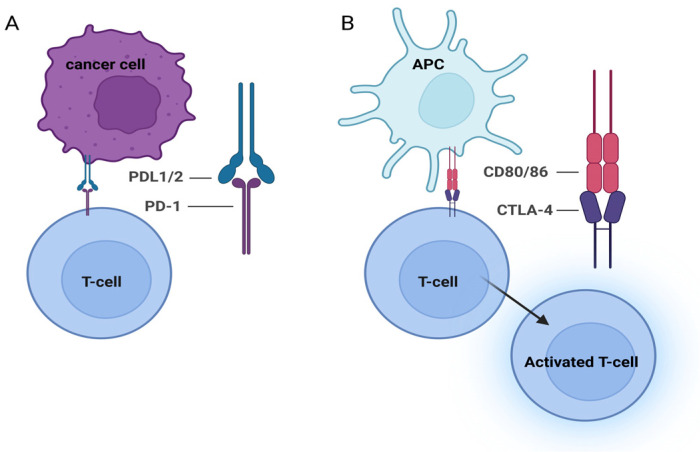
Interactions between immune checkpoint molecules and receptors on immune system cells. (**A**) Programmed cell death receptor 1 (PD-1) binding to programmed death ligand 1/2 (PD-L1/2); (**B**) cytotoxic T cell antigen 4 (CTLA-4) binding to CD80/CD86. This figure was created under the bioRENDER license.

The beneficial effect of treatment based on ICIs and chemotherapy has been demonstrated. The combination of programmed cell death protein 1 (PD-1) inhibitors with chemotherapy has been studied extensively and proved to be more efficient than chemotherapy alone. At present, cancer treatment is customised to the patient’s needs; in most cases, a combination of available methods is used [21]. Over the past 20 years, there has been a marked increase in knowledge regarding immune checkpoints in the prevention of autoimmunisation. Checkpoints regulate the stimulation of T cells at many levels of the immune response [22]. Many molecules have been discovered that constitute checkpoints. Interactions between immune checkpoint molecules PD-1 or CTLA-4 and their receptors on immune system cells are presented in Figure 2. 

CTLA-4, a CD28 homologue, has not been detected in naive T cells, but appears shortly after activation of T cells [23,24]. CTLA-4 expression is stimulated as a result of the activation of the T cell receptor TCR/CD3 complex; CTLA-4 is therefore a negative feedback signal in the specific immune response, preventing its excessive development. Overproduction of CTLA-4 is associated with the presence of multiple TCR-related signalling molecules that bind to the major histocompatibility complex (MHC) antigen present on the surface of antigen-presenting cells (APCs), resulting in competition between CD28 and CTLA-4 for binding to CD80 (B7-1) and CD86 (B7-2). Compared with CD28, CTLA-4 has a much higher affinity for B7 [22,23,24]. The goal of CTLA-4–ligand binding is to minimise defects in properly functioning tissues and to prevent the development of autoimmunisation, which leads to the inhibition of the signal associated with T cell proliferation, but also to a decrease in survival and in the ability to differentiate, thus contributing to a reduction in the production of certain cytokines [22,25,26]. Interestingly, the monomeric form of CTLA-4 has the ability to bind ligands, but without activating signalling pathways. In addition, regulatory T cells (Tregs) express CTLA-4. These cells are closely related to the maintenance of the suppressive functions of lymphocytes, and their deficiency causes dysfunction [22,25]. CTLA-4 also undergoes regulatory processes that are closely related to their own distribution in the cell [22]. To summarise, CTLA-4 may play a two-fold role in disease processes: first, increased expression may lead to immunosuppression, and second, its deficiency or dysfunction leads to loss of control over lymphocytes and to the development of inflammatory diseases.

Another important control molecule is PD-1, which is homologous to the co-stimulating receptors B7 and CD28 [22,27]. PD-1 is active in a variety of cell types, including T cells, monocytes, macrophages, and B cells [27,28,29,30]. Additionally, they may bind to ligands such as programmed death ligand 1 (PD-L1) and PD-L2, significantly slowing the stimulation of the immune system and thus normalising both peripheral and central tolerance [27,28]. By contrast, PD-L1 is expressed on lymphatic, myeloid, and normal epithelial cells. The PD-1–PD-L1 co-interaction significantly enhances immune tolerance, preventing disproportionate immune system dynamics and protecting the body from autoimmunisation and unnecessary disorganisation of immune cells [31]. PD-1 restricts the proliferation of T cells, decreases interferon gamma (IFNγ) production, and reduces T cell survival. When a T cell binds to both TCR and PD-1, the signalling mediated by PD-1 blocks the phosphorylation of transient products, which eventually inhibits the initial TCR stimuli, leading to a reduction in T cell activation [22,29]. Although anti-PD-1 treatment showed promise compared with placebo in patients with GC in a phase 3 trial, it failed when compared with chemotherapy [32]. Like CTLA-4, PD-1 exerts negative effects on T cells, but the mechanisms of action are different. CTLA-4 is restricted to T cells only, while PD-L1 is found on both T and B cells and on myelogenous cells. They also act at different times: CTLA-4 at the initial stage of T cell stimulation and PD-1 at the effector stage and primarily in peripheral tissues [30].

The CTLA-4 and PD-1 signalling pathways play important roles in maintaining homeostasis and transmit many signals involved in cancer [27]. CTLA-4 is also expressed in cancer cells, which affects the development of haematological and solid tumours. At the initial stage of carcinogenesis, T cell stimulation by CTLA-4 is usually minimised. This is due to the generation of stimuli that slow down the process, leading to a significant reduction in the immune response to the cancer. In addition, CTLA-4 contributes to reduce T cell proliferation [33]. On the other hand, the PD1–PD-L1 signalling pathway provides an ideal way for cancer cells to escape the immune response [27,31]. Cancer cells activate this pathway by using inflammatory cytokines such as IFNγ and specific tumour signalling pathways. In addition, tumour-infiltrating lymphocytes (TILs) and cells located within the tumour framework can express PD-L1, which causes a pronounced T cell deficiency that favours the formation of an immunosuppressive environment and supports tumour progression [22,34]. 

### 3.2. Importance of Immune Checkpoint Inhibitors in Cancer Therapy

**Figure 3:**
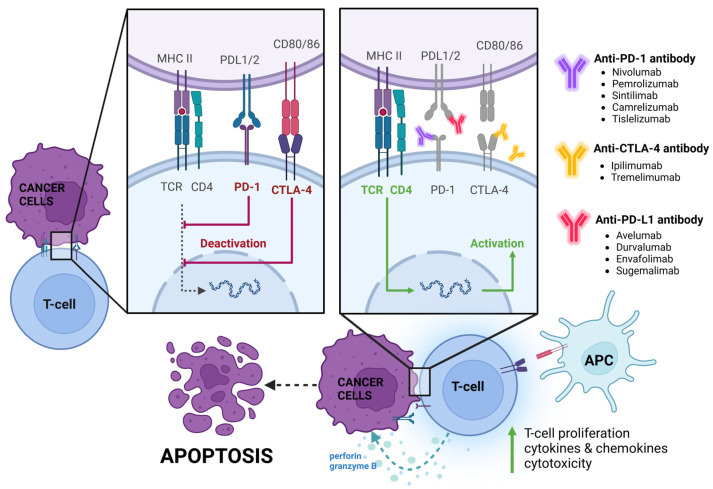
Mechanism of blocking immune checkpoint molecules in gastric cancer (GC). This figure was created under the bioRENDER license. Abbreviations: CTLA-4, cytotoxic T cell antigen 4; MHC, major histocompatibility complex; PD-1, programmed cell death receptor 1; PD-L1, programmed death ligand 1.

Immune checkpoint molecules (ICPMs) are proteins that modulate innate and adaptive immune responses [35,36]. Their expression is prominent on immune system cells, mainly APCs, as well as on tumour cells, while the specific ligands are expressed mainly on immune cells [35,36]. The engagement of the receptor by the ligand is crucial for producing the inhibitory or stimulatory signal in the immune cell [35,36]. The mechanism of action of ICIs is shown in Figure 3. Their natural role is to prevent the immune system from overreacting and to maintain homeostasis during antimicrobial and antiviral responses [37]. The vast majority of well-known and effectively targeted ICPMs are expressed by T cells, but the cells of the innate immune system can also contribute, underscoring the complex nature of the process [38]. 

There are three different groups of ICIs that have been approved by the U.S. Food and Drug Administration (FDA) for many types of cancer, including PD-1 inhibitors (e.g., nivolumab, pembrolizumab, and cemiplimab), PDL-1 inhibitors (e.g., atezolizumab, durvalumab, and avelumab), and a CTLA-4 inhibitor (ipilimumab), which have been widely used in the last decade [39]. For several years, some of the ICIs have been for cancer immunotherapy due to the observed immune dysregulation caused by the disease, including upregulation of Tregs, M2 macrophages, and cytokines, effectively impacting the tumour microenvironment [40,41]. T cells—mainly cytotoxic T cells (CTLs), Tregs, and Th1 cells that secrete IFNγ—are crucial for controlling tumour growth. Cytotoxicity is a complex process where activated CD8+ T cells release IFNγ to cause tumour cell death by upregulating the expression of MHC class I on tumour cells and inhibiting tumour cell proliferation [42]. Tumour cells disrupt this immune response by downregulating MHC class I expression on their cell surface, which limits immune recognition by CD8+ T cells [42]. Therefore, ICPMs reduce the suppression of T cells and simultaneously improve tumour-specific immune responses [42,43].

Tregs play a critical role in anticancer immunity. Their depletion improves antitumour T cell responses and reduces tumour growth, a phenomenon that can be mediated by ICPMs [44,45]. Indeed, ICPMs can increase the production of suppressive cytokines and promote cytolysis, metabolic arrest, and dendritic cell (DC) suppression [46,47]. Thus, targeting Tregs can be an effective approach for immunotherapy and encompasses numerous methods, such as lowering the number of Tregs, suppressing their function and disrupting Treg recruitment to the tumour microenvironment [45]. 

In summary, immunotherapy with ICPMs aims to attenuate specific ligands on tumour cells, reversing T cell exhaustion caused by cancer and restoring antitumour immunity [41]. Interestingly, this approach may also apply to several chronic viral infections caused by the human immunodeficiency virus (HIV), hepatitis B virus (HBV), hepatitis C virus (HCV), herpes simplex virus (HSV), EBV, varicella–zoster virus (VZV), cytomegalovirus, and, recently, severe acute respiratory syndrome coronavirus 2 (SARS-CoV-2) [48]. It is worth noting that ICPMs are promising prognostic and preventive biomarkers in many cancers [49].

Some key ICPMs, including CTLA-4, indoleamine 2,3-dioxygenase (IDO), lymphocyte activation gene 3 protein (LAG-3), T cell immunoglobulin and mucin domain-containing protein 3, and PD-1, are often overexpressed in immune cells from patients with GC [50,51,52]. Moreover, tumour-induced T cell exhaustion can promote GC progression [53]. Overall, patients with GC tolerate ICI therapy better than chemotherapy regimens, although the side effects that occur are broadly similar—decreased appetite, diarrhoea, nausea, pruritus or rash, and general fatigue [54,55,56]—but they occur during the first or second infusions. Anti-PD-1/PD-L1 antibodies have shown positive clinical activity in advanced GC as well as in gastroesophageal junction cancer (Table 1).

Results from clinical trials are needed to further evaluate the potential roles of these agents [53]; in the next section we have therefore collected all currently active or completed studies conducted using ICIs that are registered and available on the ClinicalTrials.gov website.

## 4. Clinical Trials That Have Evaluated Immune Checkpoint Inhibitors in Gastric Cancer

The primary goal regarding GC is to improve the quality of the diagnosis and treatment of patients with this disease. There are numerous approved therapies to treat the disease, and the main goal of new clinical trials is to find a cure for the disease or to improve the patient’s well-being and quality of life. Each new clinical trial must have specific patient admission criteria and be conducted in accordance with the protocol and established principles. Patients enrolled in clinical trials must meet specific criteria and follow certain requirements. Clinical trials provide an answer regarding whether the proposed treatment is more effective and better tolerated than currently used treatments and whether it can or should be routinely used in medicine. This is very important because it gives hope, especially to patients with advanced forms of GC that are difficult to treat. Participation in such clinical trials can provide patients with access to novel therapies years before they become available as standard treatment.

As of 2024, 59 clinical trials examining ICIs in GC have been registered at clinicaltrials.gov (Table 2). Of these, six have been completed, 13 are active (not recruiting), 28 are still recruiting, and one is enrolling by invitation. One has been withdrawn, four have terminated, and six have an unknown status (they are not included in Table 2). Some of these trials focus on the use of a single ICI, and some evaluate multiple ICIs used in combination or with other drugs.

Extensive clinical trials have successfully revealed that ICIs exhibit favourable GC therapeutic effects [57]. For Her2-positive patients, trastuzumab with first-line chemotherapy plus pembrolizumab has shown beneficial response rates and has been approved for use in the USA (KEYNOTE-811). Results described by Janjigian et al. [58] showed that adding pembrolizumab to trastuzumab and chemotherapy induced complete responses in some patients, significantly decreased tumour size, and improved the objective response rate (ORR). For the group of Her2-positive patients who did not benefit from trastuzumab or were progressive under prior trastuzumab therapy, some innovative approaches of Her2-directed therapy are under investigation. These include T-DXd (antibody-drug conjugate trastuzumab–deruxtecan) [59], which was approved in 2021 by the FDA as a second Her2-directed therapy option for patients with unresectable, locally advanced or metastatic GC. T-DXd is currently under investigation as a combination therapy with ICIs.

In a third-line therapy based on nivolumab, the superior overall survival (OS) increased (approval in Japan), and pembrolizumab showed a positive effect on the duration of response (KEYNOTE-059). It should be emphasised that nivolumab is the first PD-1 inhibitor approved for advanced GC patients as a third-line treatment. The safety and effectiveness of nivolumab as a single agent and in combination with ipilimumab in advanced solid tumours have been evaluated in phase I/II clinical trials. The results of the KEYNOTE-590 study based on patients with advanced GEJC and EC showed good results for a combination of pembrolizumab plus chemotherapy in Europe (CPS ≥ 10) and in the USA. An analysis of KEYNOTE-059, KEYNOTE-061, and KEYNOTE-062 demonstrated that pembrolizumab was more effective than chemotherapy as a first-line treatment. Unfortunately, a significant number of GC patients whose disease progressed after first- and second-line therapy determined third- and last-line treatment to be less advisable options. The results of a systematic review and meta-analysis of randomised controlled trials indicated that third- and later-line therapies were more effective in advanced GC patients [60]. Despite many toxic effects in third-line treatment regimens, their safety profile encourages the use of single or combined immunotherapy, even in later lines of treatment. 

These clinical trials successfully revealed that ICIs produce favourable GC therapeutic effects [57].

## 5. Future Outlook and Conclusions

In recent years, therapy based on blocking immune system checkpoints and thereby activating an immune response has shown great effectiveness in the treatment of various cancers, including GC. The use of immunocompetent molecules is already standard practice in the treatment of many cancers, including melanoma, lung cancer, kidney cancer, colon cancer, and head and neck cancer. FDA-approved immune checkpoint inhibitors include anti-PD-1 antibodies pembrolizumab, nivolumab, and cemiplimab, anti-CTLA-4 antibody ipilimumab, and anti-PD-L1 antibodies atezolizumab, avelumab, and durvalumab. Treatment of gastrointestinal cancer requires a comprehensive approach and efficient, high-quality molecular and genetic diagnostics to ensure patients receive optimal care. A major breakthrough in immunotherapy for advanced gastric cancer (AGC) was the approved PD-1 monoclonal antibody for third-line treatment, and PD-1 inhibitors such as nivolumab and pembrolizumab have already been approved in monotherapy and in combination therapy for advanced EGAC in first- or third-line settings in Europe, the USA, and Asia [60]. PD-1 inhibitors have also been approved for the first-line treatment of patients with AGC, gastroesophageal junction cancer, and esophageal adenocarcinoma. However, the results of several clinical trials are not entirely consistent. Interestingly, GC patients with a CPS ≥ 10 received a more significant benefit [61].

It should be remembered that the efficacy of the use of immune checkpoint inhibitors alone is limited; therefore, the combination of the PD-1 monoclonal antibody and chemotherapy (cisplatin, 5-FU) has now become the new standard for the first-line treatment of AGC [62]. Despite increasingly better diagnostics and modern therapy, the 5-year survival rate for advanced GC is still less than 10%, and the median overall survival is still less than 1 year. Patients diagnosed late, with advanced disease, or with other conditions that may adversely respond to standard GC therapies (including chemotherapy and radiotherapy) have limited options and poorer treatment standards. Our body has mechanisms to fight cancer, but unfortunately malignant cells can escape and evade immune elimination. Hence, we urge researchers to gain a deeper understanding of how to safely direct the patient’s immune system cells to fight cancer cells. Of note, ICPMs represent promising prognostic and predictive biomarkers in many cancers. Each subsequent clinical trial for immunotherapy or targeted therapy has produced increasingly better treatment results for patients with gastrointestinal cancer, even in the advanced stages of the disease. Immunotherapy in the neoadjuvant and adjuvant settings as well as in the second- and later-line treatment of late-stage gastrointestinal cancers has demonstrated surprising but promising potential. 

Of course, like any other therapy, immunotherapy based on the use of ICIs is associated with a number of side effects that may be observed in patients. The most serious side effects associated with treatment are the death of the patient, caused by severe toxicity of a combined treatment or an aggressive reaction of the immune system, including a cytokine storm. Particularly severe side effects have been reported among patients receiving both chemotherapy and anti-PD-1 or anti-PD-L1 inhibitors [63,64]. It should be remembered that the key to an effective GC treatment is a “teamwork” approach utilising various existing therapies, such as immunotherapy in addition to adjuvant and neoadjuvant protocols. ICIs as well as chimeric antigen receptor (CAR)-T cell therapies are predicted to have the greatest potential for further improving the prognosis for cancer patients. Interestingly, the use of ICIs has been shown to yield better clinical results than CAR-T cells in treating solid tumours [65].

The greatest challenges associated with the routine use of ICPM in therapy include (apart from the side effects of its use) its limitations, such as non-effective immunotherapy or immunotherapy resistance, which reduce the effectiveness of the treatment. Why does immunotherapy not always work? The patient’s response to immune therapy depends on multiple factors that may be responsible for immunoresistance, i.e., factors closely related to the patient’s health and lifestyle, such as genomic factors, factors related to immune system cells or to the gastric cancer microenvironment, factors emerging from the host cells, as well as advanced age, biological sex, diet, hormones, existing comorbidities, or even the composition of the gut microbiome. It should be noted that GC has a complex tumour microenvironment; there are therefore differences in GC patients’ epidemiological characteristics, clinicopathological features, biological behaviour, therapeutic modes, as well as drug selections between Eastern and Western populations. Therefore, selecting the appropriate group of patients for clinical trials and then drawing conclusions and formulating recommendations is crucial. Another problem involved in stopping the development of gastric cancer (as well as other types of cancer) is limiting the process of angiogenesis, which is responsible for the vascularisation of the tumour, thus opening the way to metastasis. Therefore, the next challenge and therapeutic goal will be to combine ICI therapy with anti-angiogenic therapy. Early results from the use of ramucirumab in combination with anti-PD-1/PD-L1 therapy are promising options for improving patient survival [66,67]. It seems that searching for a therapy based on combining ICIs with other ICIs, anti-VEGF agents and radiotherapy will be the plan for the next few years in the treatment of GC. Hyperprogression is another challenging phenomenon associated with the use of immune checkpoint inhibitors as a form of immunotherapy. This phenomenon is characterised by unexpectedly rapid disease progression in response to the immunotherapy drug administration, even faster than it probably would have progressed without any medication. There are some genetic factors warranting further research, but no confirmed molecular defects known among patients with hyperprogression after immunotherapy [68].

In short, we have various forms of cancer immunotherapy other than ICIs that we can select appropriate to the patient’s health, such as cytokine therapies, oncolytic virus therapies, cancer vaccines, and adoptive cell transfer. Progress in GC clinical practice will come when we appropriately combine different treatment strategies and select a therapy tailored to each patient. Obviously, ICIs have completely transformed cancer immunotherapy, and future studies that explore new ICI drug combination strategies for patients with GC are needed. These results will be scrutinised carefully. 

## Figures and Tables

**Table 1:** Immune checkpoint molecules (ICPMs) and their functions.

ICPM	Cells Expressing the ICPM	Ligand/Receptor	Function
PD-1	T cells	PD-L1/PD-L2	Interaction between PD-1 and ligands and impacts cytokine secretion
PD-L1 (CD274, B7H1)	DCs and macrophages	PD-1	Inhibits T cell responses by anergising tumour-reactive T cells by binding to its PD-1 receptor; renders tumour cells resistant to CD8+ T cell and Fas ligand–mediated lysis; tolerises T cells through CD80
CTLA-4	Tregs	CD80/86	Inhibits T cell responses
BTLA (CD272)	T CD8+ and T CD4+, NK cells, B cells, DCs, and macrophages	unknown	Inhibits T cell responses and maintains immune homeostasis
B7H3 (CD276)	T and B-cells, monocytes, DCs, MDSCs, neutrophils, and macrophages	unknown	Inhibits T cell responses and proliferation, and downregulates cytokine production
B7H4 (B7x, B7S1)	T cells, B cells, monocytes, and DCs	unknown	Inhibits T cell proliferation, cell cycle progression, and cytokine production
HHLA2 (B7H5, B7H7)	APCs, monocytes, B cells and DCs	unknown	Inhibits T cells
IOD1	T cells and NK cells	unknown	Suppresses CD8+ T cells and NK cells, and induces iTregs
PVRIG	DCs, Th1, and NK cells	CD112	Inhibits T cell responses
TIM-3	DCs, NK cells, Th1 cells, Th17 cells, and macrophages	GAL-9, PS	Inhibits T cell responses
GAL-9	Eosinophils, DCs, IEC, T cells, macrophages, lymphoid cells, Kupffer cells, and vascular endothelial cells	TIM-3	Maintains immune homeostasis
VISTA	T cells and APCs	unknown	Inhibits T cell responses
LAG3 (CD223)	Plasmacytoid DCs, NK T cells, and Tregs	MHC II, GAL-9, FGL1	Interacts with MHC II
TIGIT	T cells and NK cells	CD155, CD112	Suppresses anti-tumour immunity
CD28	T cells	CD80/CD86 (form CTLA-4)	Inhibits T cell responses
CD40	B cells, DCs, and HPCs	CD154	Activates several signalling pathways;
CD70	T cells, B cells, and DCs	CD27	Stimulates T cell differentiation, enhances cytotoxicity of T cells, and promotes TNF-α production
CD47	RBCs and non-HPCs	integrins	Inhibits macrophage activity
CD137	T cells and APCs	CD137L (TNFSF9, 4-1BBL)	Activates the MAPK and NF-κB signalling pathway

**Table 2:** Clinical trials based on immune checkpoint inhibitors in gastric cancer therapy.

Trial Number	Type of GC	Status/Phase	Checkpoint Inhibitor(s)	Age (Years)	Locations
NCT04694183	Advanced, unresectable, metastatic GC	Completed	Camrelizumab	18–75	China
NCT02903914	Metastatic/locally advanced GC	Completed	Pembrolizumab	≥18	USA, Italy, Spain, and The Netherlands
NCT04294784	Recurrent or metastatic gastric and esophagogastric adenocarcinoma	Active, not recruiting	Shr-1210	18–70	China
NCT04267549	Stage IV gastric adenocarcinoma	Active, not recruiting	Sintilimab	18–75	China
NCT03841110	Advanced solid tumours	Completed	Nivolumab, pembrolizumab, atezolizumab,	≥18	USA
NCT03321630	Metastatic or recurrent gastric or gastroesophageal junction (GEJ) adenocarcinoma	Completed	Pembrolizumab	18–100	USA
NCT06238752	HER2-negative, advanced G/GEJ cancer patients with signet ring cell carcinoma or peritoneal metastasis	Completed	Tislelizumab	≥18	China
NCT04249739	Advanced gastric or gastroesophageal junction (GEJ) adenocarcinoma–EBV negative and MSS (or MMR-proficient) GC	Active, not recruiting	Pembrolizumab	≥19	Republic of Korea
NCT04082364	HER2-positive gastric cancer (GC) or gastroesophageal junction (GEJ) cancer	Active, not recruiting	Retifanlimab, Tebotelimab (anti PD-1, anti-LAG3)	≥18	USA, China, Germany, Italy, Republic of Korea, Poland, Singapore, Taiwan, and UK
NCT03236935	Recurrent, locally advanced, or metastatic gastric cancer	Active, not recruiting	Pembrolizumab	≥18	USA
NCT04306900	Unresectable or metastatic solid tumours	Completed	Pembrolizumab, budigalimab	18–110	USA and Republic of Korea
NCT05311176	Advanced or metastatic HER2/neu overexpressing gastric or GEJ adenocarcinoma	Active, not recruiting	Pembrolizumab	≥18	Australia and Taiwan
NCT03797326	Advanced (metastatic and/or unresectable) solid tumours	Active, not recruiting	Pembrolizumab	≥18	USA, Argentina, Australia, Canada, Chile, Colombia, France, Germany, Israel, Italy, Republic of Korea, Russian Federation, Spain, Switzerland, Taiwan, Thailand and UK
NCT03228667	Recurrent locally advanced or metastatic gastric or gastroesophageal junction adenocarcinoma	Active, not recruiting	Nivolumab Pembrolizumab, Atezolizumab, Avelumab, Durvalumab	≥18	USA
NCT02465060	Advanced refractory solid tumours	Active, not recruiting	Nivolumab, Relatlimab	≥18	USA, Guam, and Puerto Rico
NCT04078152	Any type	Active, not recruiting	Durvalumab	18–130	USA, Argentina, Australia, Belgium, Brazil, Bulgaria, Canada, Chile, Czechia, France, Germany, Greece, Hungary, India, Israel, Japan, Republic of Korea, Malysia, The Netherlands, Poland, Romania, Russian Federation, Serbia, Switzerland, Spain, Taiwan, Thailand, Turkey, Ukraine, UK, and Vietnam
NCT03170960	Locally advanced or metastatic solid tumours	Active, not recruiting	Atezolizumab	≥18	USA, Australia, Belgium, France, UK, Germany, Italy, The Netherlands, and Spain
NCT03539822	Advanced gastroesophageal cancer and other gastrointestinal (GI) malignancies	Active, not recruiting	Durvalumab, Tremelimumab	≥18	USA

## References

[1] Morgan E., Arnold M., Camargo M.C., Gini A., Kunzmann A.T., Matsuda T., Meheus F., Verhoeven R.H.A., Vignat J., Laversanne M. (2022). The current and future incidence and mortality of gastric cancer in 185 countries, 2020–2040: A population-based modelling study. EClinicalMedicine.

[2] Lauren P. (1965). The two histological main types of gastric carcinoma: Diffuse and so-called intestinal-type carcinoma. An attempt at a histo-clinical classification. Acta Pathol. Microbiol. Scand..

[3] Zhang Y., Zhang P.S., Rong Z.Y., Huang C. (2021). One stomach, two subtypes of carcinoma-the differences between distal and proximal gastric cancer. Gastroenterol. Rep..

[4] Cancer Genome Atlas Research Network (2014). Comprehensive molecular characterization of gastric adenocarcinoma. Nature.

[5] Keszei A.P., Goldbohm R.A., Schouten L.J., Jakszyn P., van den Brandt P.A. (2013). Dietary N-nitroso compounds, endogenous nitrosation, and the risk of esophageal and gastric cancer subtypes in The Netherlands Cohort Study. Am. J. Clin. Nutr..

[6] Zamani N., Hajifaraji M., Fazel-tabar Malekshah A., Keshtkar A.A., Esmaillzadeh A., Malekzadeh R. (2013). A case-control study of the relationship between gastric cancer and meat consumption in Iran. Arch. Iran. Med..

[7] Yusefi A.R., Bagheri Lankarani K., Bastani P., Radinmanesh M., Kavosi Z. (2018). Risk Factors for Gastric Cancer: A Systematic Review. Asian Pac. J. Cancer Prev..

[8] Li Q., Zhang J., Zhou Y., Qiao L. (2012). Obesity and gastric cancer. Front. Biosci..

[9] Uemura N., Okamoto S., Yamamoto S., Matsumura N., Yamaguchi S., Yamakido M., Taniyama K., Sasaki N., Schlemper R.J. (2001). Helicobacter pylori infection and the development of gastric cancer. N. Engl. J. Med..

[10] Bertuccio P., Chatenoud L., Levi F., Praud D., Ferlay J., Negri E., Malvezzi M., La Vecchia C. (2009). Recent patterns in gastric cancer: A global overview. Int. J. Cancer.

[11] Machlowska J., Baj J., Sitarz M., Maciejewski R., Sitarz R. (2020). Gastric Cancer: Epidemiology, Risk Factors, Classification, Genomic Characteristics and Treatment Strategies. Int. J. Mol. Sci..

[12] Crew K.D., Neugut A.I. (2006). Epidemiology of gastric cancer. World J. Gastroenterol..

[13] Japanese Gastric Cancer Association (2023). Japanese Gastric Cancer Treatment Guidelines 2021 (6th edition). Gastric Cancer.

[14] Jiang T., Mei L., Yang X., Sun T., Wang Z., Ji Y. (2022). Biomarkers of gastric cancer: Current advancement. Heliyon.

[15] Grávalos C., Jimeno A. (2008). HER2 in gastric cancer: A new prognostic factor and a novel therapeutic target. Ann. Oncol..

[16] Wang F.H., Zhang X.T., Li Y.F., Tang L., Qu X.J., Ying J.E., Zhang J., Sun L.Y., Lin R.B., Qiu H. (2021). The Chinese Society of Clinical Oncology (CSCO): Clinical guidelines for the diagnosis and treatment of gastric cancer, 2021. Cancer Commun..

[17] Rose S. (2018). Two Win Nobel for Immune Regulation Discoveries. Cancer Discov..

[18] Leowattana W., Leowattana P., Leowattana T. (2023). Immunotherapy for advanced gastric cancer. World J. Methodol..

[19] Ariga S. (2023). History and Future of HER2-Targeted Therapy for Advanced Gastric Cancer. J. Clin. Med..

[20] Liu K., Wu C.X., Liang H., Wang T., Zhang J.Y., Wang X.T. (2024). Analysis of the impact of immunotherapy efficacy and safety in patients with gastric cancer and liver metastasis. World J. Gastrointest. Surg..

[21] Liu B.W., Shang Q.X., Yang Y.S., Chen L.Q. (2023). Efficacy and safety of PD-1/PD-L1 inhibitor combined with chemotherapy versus chemotherapy alone in the treatment of advanced gastric or gastroesophageal junction adenocarcinoma: A systematic review and meta-analysis. Front. Oncol..

[22] Buchbinder E.I., Desai A. (2016). CTLA-4 and PD-1 Pathways: Similarities, Differences, and Implications of Their Inhibition. Am. J. Cilin. Oncol..

[23] Liu F., Huang J., Liu X., Cheng Q., Luo C., Liu Z. (2020). CTLA-4 correlates with immune and clinical characteristics of glioma. Cancer Cell Int..

[24] Chikuma S. (2017). CTLA-4, an Essential Immune-Checkpoint for T-Cell Activation. Curr. Top Microbiol. Immunol..

[25] Wing K., Onishi Y., Prieto-Martin P., Yamaguchi T., Miyara M., Fehervari Z., Nomura T., Sakaguchi S. (2008). CTLA-4 control over Foxp3+ regulatory T cell function. Science.

[26] Qin S., Xu L., Yi M., Yu S., Wu K., Luo S. (2019). Novel immune checkpoint targets: Moving beyond PD-1 and CTLA-4. Mol. Cancer.

[27] Klatka J., Szkatuła-Łupina A., Hymos A., Klatka M., Mertowska P., Mertowski S., Grywalska E., Charytanowicz M., Błażewicz A., Poniewierska-Baran A. (2022). The Clinical, Pathological, and Prognostic Value of High PD-1 Expression and the Presence of Epstein–Barr Virus Reactivation in Patients with Laryngeal Cancer. Cancers.

[28] Batur S., Kain Z.E., Gozen E.D., Kepil N., Aydin O., Comunoglu N. (2020). Programmed Death Ligand 1 Expression in Laryngeal Squamous Cell Carcinomas and Prognosis. Clin. Pathol..

[29] Parry R.V., Chemnitz J.M., Frauwirth K.A. (2005). CTLA-4 and PD-1 Receptors Inhibit T-Cell Activation by Distinct Mechanisms. Mol. Cell Biol..

[30] Keir M.E., Butte M.J., Freeman G.J., Sharpe A.H. (2008). PD-1 and its ligands in tolerance and immunity. Annu. Rev. Immunol..

[31] Kythreotou A., Siddique A., Mauri F.A., Bower M., Pinato D.J. (2018). PD-L1. BMJ Publ. Group.

[32] Coutzac C., Pernot S., Chaput N., Zaanan A. (2019). Immunotherapy in advanced gastric cancer, is it the future?. Crit. Rev. Oncol. Hematol..

[33] Zhao Y., Yang W., Huang Y., Cui R., Li X., Li B. (2018). Evolving Roles for Targeting CTLA-4 in Cancer Immunotherapy. Cell. Physiol. Biochem..

[34] Zhang H., Dai Z., Wu W., Wang Z., Zhang N., Zhang L., Zeng W.J., Liu Z., Cheng Q. (2021). Regulatory mechanisms of immune checkpoints PD-L1 and CTLA-4 in cancer. J. Exp. Clin. Cancer Res..

[35] Marcucci F., Rumio C., Corti A. (2017). Tumor cell-associated immune checkpoint molecules—Drivers of Malignancy and stemness. Biochim. Biophys. Acta Rev. Cancer..

[36] Pardoll D. (2012). The blockade of immune checkpoints in cancer immunotherapy. Nat. Rev. Cancer..

[37] Akbulut Z., Aru B., Aydın F., Yanıkkaya D.G. (2024). Immune checkpoint inhibitors in the treatment of hepatocellular carcinoma. Front. Immunol..

[38] Pauken K.E., Wherry E.J. (2015). Overcoming T cell exhaustion in infection and cancer. Trends Immunol..

[39] Seidel J., Otsuka A., Kabashima K. (2018). Anti-PD-1 and Anti-CTLA-4 Therapies in Cancer: Mechanisms of Action, Efficacy, and Limitations. Front. Oncol..

[40] Shiravand Y., Khodadadi F., Kashani S.M.A., Hosseini-Fard S.R., Hosseini S., Sadeghirad H., Ladwa R., O’Byrne K., Kulasinghe A. (2022). Immune Checkpoint Inhibitors in Cancer Therapy. Curr. Oncol..

[41] Dyck L., Mills K.H.G. (2017). Immune checkpoints and their inhibition in cancer and infectious diseases. Eur. J. Immunol..

[42] Apetoh L., Smyth M.J., Drake C.G., Abastado J.P., Apte R.N., Ayyoub M., Blay J.Y., Bonneville M., Butterfield L.H., Caignard A. (2015). Consensus nomenclature for CD8 T cell phenotypes in cancer. Oncoimmunology.

[43] Blackburn S.D., Shin H., Haining W.N., Zou T., Workman C.J., Polley A., Betts M.R., Freeman G.J., Vignali D.A., Wherry E.J. (2009). Coregulation of CD8+ T cell exhaustion by multiple inhibitory receptors during chronic viral infection. Nat. Immunol..

[44] Dong P., Xiong Y., Yue J., Hanley S.J.B., Watari H. (2018). Tumor-Intrinsic PD-L1 Signaling in Cancer Initiation, Development and Treatment: Beyond Immune Evasion. Front. Oncol..

[45] Nair V.S., Elkord E. (2018). Immune checkpoint inhibitors in cancer therapy: Ocus on T—Regulatory cells. Immunol. Cell Biol..

[46] Vignali D.A., Collison L.W., Workman C.J. (2008). How regulatory T cells work. Nat. Rev. Immunol..

[47] Marcucci F., Rumio C. (2021). Depleting tumor cells expressing immune checkpoints ligands—A new approach to combat cancer. Cells.

[48] Niedźwiedzka-Rystwej P., Majchrzak A., Aksak-Wąs B., Serwin K., Czajkowski Z., Grywalska E., Korona-Głowniak I., Roliński J., Parczewski M. (2022). Programmed Cell Death-1/Programmed Cell Death-1 Ligand as Prognostic Markers of Coronavirus Disease 2019 Severity. Cells.

[49] Ballman K.V. (2015). Biomarker: Predictive or prognostic?. J. Clin. Oncol..

[50] Kim J.W., Nam K.H., Ahn S.H., Park D.J., Kim H.H., Kim S.H., Chang H., Lee J.O., Kim Y.J., Lee H.S. (2016). Prognostic implications of immunosuppressive protein expression in tumors as well as immune cell infiltration within the tumor microenvironment in gastric cancer. Gastric Cancer.

[51] Takaya S., Saito H., Ikeguchi M. (2015). Upregulation of Immune Checkpoint Molecules, PD-1 and LAG-3, on CD4+ and CD8+ T Cells after Gastric Cancer Surgery. Yonago Acta Med..

[52] Cheng G., Li M., Wu J., Ji M., Fang C., Shi H., Zhu D., Chen L., Zhao J., Shi L. (2015). Expression of Tim-3 in gastric cancer tissue and its relationship with prognosis. Int. J. Clin. Exp. Pathol..

[53] Taieb J., Moehler M., Boku N., Ajani J.A., Yañez Ruiz E., Ryu M.H., Guenther S., Chand V., Bang Y.J. (2018). Evolution of checkpoint inhibitors for the treatment of metastatic gastric cancers: Current status and future perspectives. Cancer Treat. Rev..

[54] Chung H.C., Arkenau H.T., Lee J., Rha S.Y., Oh D.Y., Wyrwicz L., Kang Y.K., Lee K.W., Infante J.R., Lee S.S. (2019). Avelumab (anti-PD-L1) as first-line switch-maintenance or second-line therapy in patients with advanced gastric or gastroesophageal junction cancer: Phase 1b results from the JAVELIN Solid Tumor trial. J. Immunother. Cancer..

[55] Kang Y.K., Boku N., Satoh T., Ryu M.H., Chao Y., Kato K., Chung H.C., Chen J.S., Muro K., Kang W.K. (2017). Nivolumab in patients with advanced gastric or gastro-oesophageal junction cancer refractory to, or intolerant of, at least two previous chemotherapy regimens (ONO-4538-12, ATTRACTION-2): A randomised, double-blind, placebo-controlled, phase 3 trial. Lancet.

[56] Muro K., Chung H.C., Shankaran V., Geva R., Catenacci D., Gupta S., Eder J.P., Golan T., Le D.T., Burtness B. (2016). Pembrolizumab for patients with PD-L1-positive advanced gastric cancer (KEYNOTE-012): A multicentre, open-label, phase 1b trial. Lancet Oncol..

[57] Wang F.H., Zhang X.T., Tang L., Wu Q., Cai M.Y., Li Y.F., Qu X.J., Qiu H., Zhang Y.J., Ying J.E. (2024). The Chinese society of clinical oncology (CSCO): Clinical guidelines for the diagnosis and treatment of gastric cancer, 2023. Cancer Commun..

[58] Janjigian Y.Y., Kawazoe A., Yañez P., Li N., Lonardi S., Kolesnik O., Barajas O., Bai Y., Shen L., Tang Y. (2021). The KEYNOTE-811 trial of dual PD-1 and HER2 blockade in HER2-positive gastric cancer. Nature.

[59] Ricci A.D., Rizzo A., Rojas Llimpe F.L., Di Fabio F., De Biase D., Rihawi K. (2021). Novel HER2-directed treatments in advanced gastric carcinoma: AnotHER paradigm shift?. Cancers.

[60] Moehler M., Högner A., Wagner A.D., Obermannova R., Alsina M., Thuss-Patience P., van Laarhoven H., Smyth E. (2022). Recent progress and current challenges of immunotherapy in advanced/metastatic esophagogastric adenocarcinoma. Eur. J. Cancer.

[61] Fei S., Lu Y., Chen J., Qi J., Wu W., Wang B., Han Y., Wang K., Han X., Zhou H. (2023). Efficacy of PD-1 Inhibitors in First-Line Treatment for Advanced Gastroesophageal Junction and Gastric Cancer by Subgroups: A Systematic Review and Meta-Analysis. Chemotherapy.

[62] Entezam M., Sanaei M.J., Mirzaei Y., Mer A.H., Abdollahpour-Alitappeh M., Azadegan-Dehkordi F., Bagheri N. (2023). Current progress and challenges of immunotherapy in gastric cancer: A focus on CAR-T cells therapeutic approach. Life Sci..

[63] Zhou X., Yao Z., Bai H., Duan J., Wang Z., Wang X., Zhang X., Xu J., Fei K., Zhang Z. (2021). Treatment-Related Adverse Events of PD-1 and PD-L1 Inhibitor-Based Combination Therapies in Clinical Trials: A Systematic Review and Meta-Analysis. Lancet Oncol..

[64] Wang Y., Zhou S., Yang F., Qi X., Wang X., Guan X., Shen C., Duma N., Vera Aguilera J., Chintakuntlawar A. (2019). Treatment-Related Adverse Events of PD-1 and PD-L1 Inhibitors in Clinical Trials. JAMA Oncol..

[65] Jia Y., Liu L., Shan B. (2020). Future of Immune Checkpoint Inhibitors: Focus on Tumor Immune Microenvironment. Ann. Transl. Med..

[66] Zeng D., Li M., Zhou R., Zhang J., Sun H., Shi M., Bin J., Liao Y., Rao J., Liao W. (2019). Tumor microenvironment characterization in gastric cancer identifies prognostic and immunotherapeutically relevant gene signatures. Cancer Immunol. Res..

[67] Mishra R., Patel H., Alanazi S., Kilroy M.K., Garrett J.T. (2021). PI3K inhibitors in cancer: Clinical implications and adverse effects. Int. J. Mol. Sci..

[68] Champiat S., Dercle L., Ammari S., Massard C., Hollebecque A., Postel-Vinay S., Chaput N., Eggermont A., Marabelle A., Soria J.-C. (2017). Hyperprogressive Disease Is a New Pattern of Progression in Cancer Patients Treated by Anti-PD-1/PD-L1. Clin. Cancer Res..

